# Cyclophilin B serum levels present variations across the menstrual cycle

**DOI:** 10.1038/s41598-023-37322-7

**Published:** 2023-06-22

**Authors:** Rebeca Alvariño, Cristina Gil-Mouce, Manuel A. Botana, Sandra Gegunde, Jesús González-Jartín, Mercedes R. Vieytes, Amparo Alfonso, Luis M. Botana

**Affiliations:** 1 Departamento de Farmacología, Facultad de Veterinaria, Universidad de Santiago de Compostela, 27002 Lugo, Spain; 2 Grupo Investigación Biodiscovery, IDIS, 27002 Lugo, Spain; 3 Departamento de Endocrinología y Nutrición, Hospital Universitario Lucus Augusti, 27002 Lugo, Spain; 4 Departamento de Fisiología, Facultad de Veterinaria, Universidad de Santiago de Compostela, 27002 Lugo, Spain

**Keywords:** Endocrinology, Molecular medicine

## Abstract

Cyclophilins are a family of chaperones involved in inflammation and cell death. Cyclophilin B is released by inflammatory cells and acts through the receptor CD147, affecting matrix metalloproteases release, whilst cyclophilin D participates in hypoxia-induced apoptosis. Previous studies related hormones like estradiol or prolactin to these proteins, however, their blood concentrations across the menstrual cycle have not been determined. In this work, eleven healthy women (BMI: 21.8 kg/m^2^) were monitored during a single menstrual cycle, making blood extractions at follicular, periovulatory and mid-luteal phases. Hormone and cyclophilin levels were determined in each phase. Statistical differences were determined by repeated measures ANOVA and estimated marginal means tests, or by Friedman and Dunn-Bonferroni tests for parametric and non-parametric variables, respectively. Bivariate correlations were evaluated with the Spearman coefficient. Cyclophilin B concentrations presented significant differences during the menstrual cycle (*p* = 0.012). The highest levels of this protein were found at follicular extraction, followed by a decrease at periovulatory phase and a slight increase at mid-luteal phase. Cyclophilin D showed the same profile, although statistical significance was not reached. This immunophilin exhibited a positive correlation with luteinizing hormone at periovulatory phase (r = 0.743, *p* = 0.009) and with follicle stimulating hormone at mid-luteal phase (r = 0.633, *p* = 0.036). This is the first study describing the changes in cyclophilin B concentrations across the menstrual cycle, as well as the association of luteinizing and follicle stimulating hormones with cyclophilin D. These results suggest a role of these proteins in the cyclic inflammatory events that affect female reproductive system that should be explored.

Cyclophilins (Cyps) are a family of immunophilins with peptidyl prolyl isomerase activity that share their affinity for the immunosuppressant drug cyclosporine A. More than 18 isoforms have been described, being cyclophilin A (CypA), cyclophilin B (CypB) and cyclophilin D (CypD) the best characterized proteins. These enzymes are involved in several intracellular pathways, such as endoplasmic reticulum homeostasis or apoptosis^1^. CypB is released to the extracellular milieu by several inflammatory cells like lymphocytes, monocytes or endothelial cells and have chemotactic and inflammatory properties mediated by CD147 activation. Regarding CypD, this protein plays a key role in cell death because it is the main regulator of mitochondrial permeability transition pore opening^2^.

Binding of extracellular CypB to CD147 leads to the activation of signalling pathways like extracellular signal-regulated kinase or the nuclear factor kappa light chain enhancer of activated B cells^3^. Activation of the receptor also produces the secretion of matrix metalloproteinases, which are primarily responsible for endometrial breakdown^4^. In this sense, CD147 presents a cyclic expression pattern across the menstrual cycle that depends on the cell type^5^. In stromal cells, CD147 levels are increased by progesterone, but uterine epithelial cells present higher levels of CD147 in the proliferative phase, which decrease in secretory and menstrual phases^5–7^.

Despite the role of Cyps in CD147 activation and cell death, their function in menstrual cycle and their association with hormones has been poorly studied. It was recently described that CypB release by spiral arterial smooth cells causes apoptosis of uterine endothelial cells, thus participating in decidualization during pregnancy^8^. Moreover, CypD inhibition reduces cell death and protects endometrial cells from oxygen deprivation^9^. On the other hand, hormones like estradiol and thyroid-stimulating hormone (TSH) were associated to an increased susceptibility of mitochondria to mPTP opening related to CypD^10–12^. In the case of CypB, it forms a transcriptional complex with prolactin that affects the expression of genes involved in cell proliferation in breast cancer^13,14^. However, the blood levels of Cyps B and D during the menstrual cycle have not been studied so far. In a previous work, in which we measured the serum concentrations of Cyps A, B, C and D in coronary artery disease patients, we observed great differences in CypB and CypD levels between men and woman in the control group, suggesting that sex hormones could be involved in the blood concentrations of these Cyps^15–17^. These results, together to the role of Cyps in inflammation, CD147 modulation and cell death, led us to hypothesize that menstrual cycle could be affecting to blood levels of Cyps B and D.

The aim of this study was to investigate the levels of CypB and CypD in serum across a single regular menstrual cycle. With this objective, the concentrations of these Cyps were monitored in follicular, periovulatory and mid-luteal phases and their relationship with sex hormones was explored.

## Methods

### Study population

The study sample consisted of 13 healthy woman between 25 and 36 years old with body mass index (BMI) in a normal range (21.8 kg/m^2^). Inclusion in the study was based on the following criteria: regular menstrual cycles (26–30 days) in the last six months, normal bleeding volume and duration of menstruation of 3–4 days. Exclusion criteria included taking hormonal contraceptives, pregnancy, breastfeeding, tobacco use and history of chronic diseases. None of the participants reported dieting or intensive physical exercise.

### Blood sampling protocol and biochemical and hormonal analysis

Blood samples were collected during follicular (cycle day 3–9), periovulatory (cycle day 14–16) and mid-luteal (cycle day 18–23) phases of a single menstrual cycle of each woman^18^. Peripheral blood was obtained from the antecubital vein using anti-coagulant free tubes (BD vacutainer®, Madrid, Spain). Samples were allowed to clot for 20 min at room temperature before centrifugation (3000 rpm for 10 min at 4 °C). Serum supernatants were collected and stored at − 80 °C until analysis.

Glucose, total cholesterol, high-density lipoprotein (HDL) and triglycerides (TG) were analysed by spectrophotometry in the automatic analyser Advia Chemistry XP (Siemens Healthineers, Madrid, Spain). Estradiol, progesterone and free thyroxine (FT4) were determined using a competitive chemiluminescent enzymatic immunoassay, whilst follicle-stimulating hormone (FSH), luteinizing hormone (LH), TSH and prolactin concentrations were measured with an ELISA sandwich in the equipment Advia Centaur XP (Siemens Healthineers).

### Measurement of cyclophilins B and D serum levels

Concentrations of CypB and CypD in serum were determined with Human Cyclophilin B ELISA kit (Catalog number: CSB-E11218h, Cusabio, Houston, US) and Human Cyclophilin D ELISA kit (Catalog number: E-EL-H1936, Elabscience, Madrid, Spain), following manufacturer’s instructions^16^. Absorbance was measured at 450 nm in a Synergy 4 microplate reader (BioTek instruments, Vermont, US). Samples were run by duplicate and concentrations were calculated using a standard curve with a range of 31.25–2000 pg/mL for CypB and 62.5–4000 pg/mL for CypD. Levels below the lower limit of determination were considered as 0 pg/mL. The intra and inter-assay coefficients of variation of the ELISA kits were < 10% and no cross reactivity was observed between Cyps^16^.

### Statistical analysis

All statistical analysis were performed with SPSS Statistics 26 for Windows. Descriptive statistics were calculated for demographics, biochemical parameters, hormone levels and Cyps concentrations. Normal distribution of variables was evaluated with Kolmogorov–Smirnov and Shapiro–Wilk tests. Data of variables with normal distribution are shown as mean ± SEM, whilst results of non-parametric variables are presented as median and interquartile range. Statistical differences across the cycle of variables with normal distribution were determined with repeated measures ANOVA and estimated marginal means (adjusted by Bonferroni method) tests. Friedman test and Dunn-Bonferroni post hoc test were used for non-parametric variables. Bivariate correlations between Cyps and hormone concentrations were determined with Spearman rank correlation coefficient. Significance level of *p* < 0.05 was set for all analyses.

### Ethical approval and consent to participate

Informed consent was obtained from all subjects and/or their legal guardian(s) prior to the inclusion in the study, which was approved by the institutional and regional ethical board (Comité Autonómico de Ética da Investigación de Galicia, Comité Territorial de Ética da Investigación de Santiago-Lugo, Secretaria Xeral, Consellería de Sanidade, Xunta de Galicia, Ref: 2019/639, according to the principles outlined in the Declaration of Helsinki).

## Results

Two participants of the initial cohort (n = 13) were excluded because they presented anovulatory cycles, without LH peak at periovulatory phase and progesterone levels below 5 ng/mL in the mid-luteal phase^19^. Woman included in the study (n = 11) had a mean age of 29.0 ± 1.2 years and presented a normal BMI (21.8 (20.3–22.6) kg/m^2^) (Table 1).

**Table 1:** Demographic and baseline characteristics of participants, biochemical parameters and hormone and cyclophilin serum levels across one menstrual cycle. Variables with normal distribution presented as mean ± SEM and non-parametric variables shown as median and interquartile range (n = 11). Significant level set at *p < 0.05 and **p < 0.01. Statistical comparisons among phases were analysed by repeated measures ANOVA test for variables with normal distribution and Friedman test for non-parametric variables.

Demographic characteristics				
Age (years)	29.0 ± 1.2			
BMI (kg/m^2^)	21.8 (20.3–22.6)			
Cyclophilin levels	Follicular phase	Periovulatory phase	Mid-luteal phase	*P* value
CypB (pg/mL)	429.0 (167.4–555.9)	214.0 (32.9–421.0)	239.9 (154.9–546.9)	0.012*
CypD (pg/mL)	692.2 (9.0–836.3)	581.3 (1.31–847.1)	690.9 (13.6–803.9)	0.097
Hormone levels				
TSH (mU/L)	3.3 ± 0.4	3.3 ± 0.4	3.5 ± 0.3	0.721
FT4 (ng/dL)	1.2 ± 0.05	1.2 ± 0.05	1.2 ± 0.05	0.481
LH (U/L)	5.2 (4.5–6.7)	12.6 (6.4–26.9)	3.8 (1.2–7.8)	0.001**
FSH (U/L)	7.9 (7.0–8.6)	8.6 (4.0–11.8)	2.5 (2.2–5.7)	0.007**
Estradiol (pg/mL)	47.8 (35.0–67.9)	107.0 (80.8–293.0)	138.7 (104.2–179.4)	0.005**
Prolactin (ng/mL)	17.1 ± 1.5	15.9 ± 1.5	19.5 ± 3.2	0.503
Progesterone (ng/mL)	0.6 (0.6–0.8)	1.4 (0.9–3.0)	8.9 (6.9–13.5)	0.002**
Hemogram and biochemical parameters				
Haemoglobin (g/dL)	13.7 ± 0.3	13.3 ± 0.3	13.5 ± 0.3	0.191
Leucocytes (number/µL)	6165.4 ± 255.1	6736.2 ± 262.6	6390.9 ± 371.1	0.246
Neutrophils (number/µL)	3500.0 ± 200.4	3936.0 ± 199.2	3445.5 ± 199.1	0.235
Lymphocytes (number/µL)	1972.7 ± 131.4	2140.3 ± 139.2	2161.5 ± 212.9	0.293
Platelets (number/µL)	231,076.9 ± 10,669.6	219,846.2 ± 10,066.35	221,461.5 ± 11,389.7	0.332
Glucose (mg/dL)	87.5 ± 1.6	81.7 ± 2.0	84.9 ± 2.3	0.018*
Total cholesterol (mg/dL)	186.2 ± 8.1	183.8 ± 7.3	182.5 ± 8.9	0.767
HDL (mg/dL)	75.6 ± 3.9	77.9 ± 3.4	76.3 ± 4.1	0.485
LDL (mg/dL)	95.5 ± 6.1	91.2 ± 7.3	92.1 ± 7.8	0.471
TG (mg/dL)	74.0 ± 5.5	66.1 ± 5.7	70.5 ± 5.1	0.237

BMI, body mass index; HDL, high-density lipoprotein; LDL, low-density lipoprotein; TG, triglycerides; TSH, thyroid-stimulating hormone; FT4, free thyroxine; LH, luteinizing hormone; FSH, follicle-stimulating hormone; CypB, cyclophilin B; CypD, cyclophilin D.

Significant values are shown in bold.

A complete biochemical analysis was performed in each phase of the menstrual cycle in order to analyse the general condition of the volunteers. Glucose levels presented significant differences across the cycle (*p* = 0.018), with a significant decrease in periovulatory phase (*p* = 0.023, compared to follicular phase), followed by an augmentation in mid-luteal extraction. No differences were detected in the rest of the parameters monitored (Table 1).

**Figure 1:**
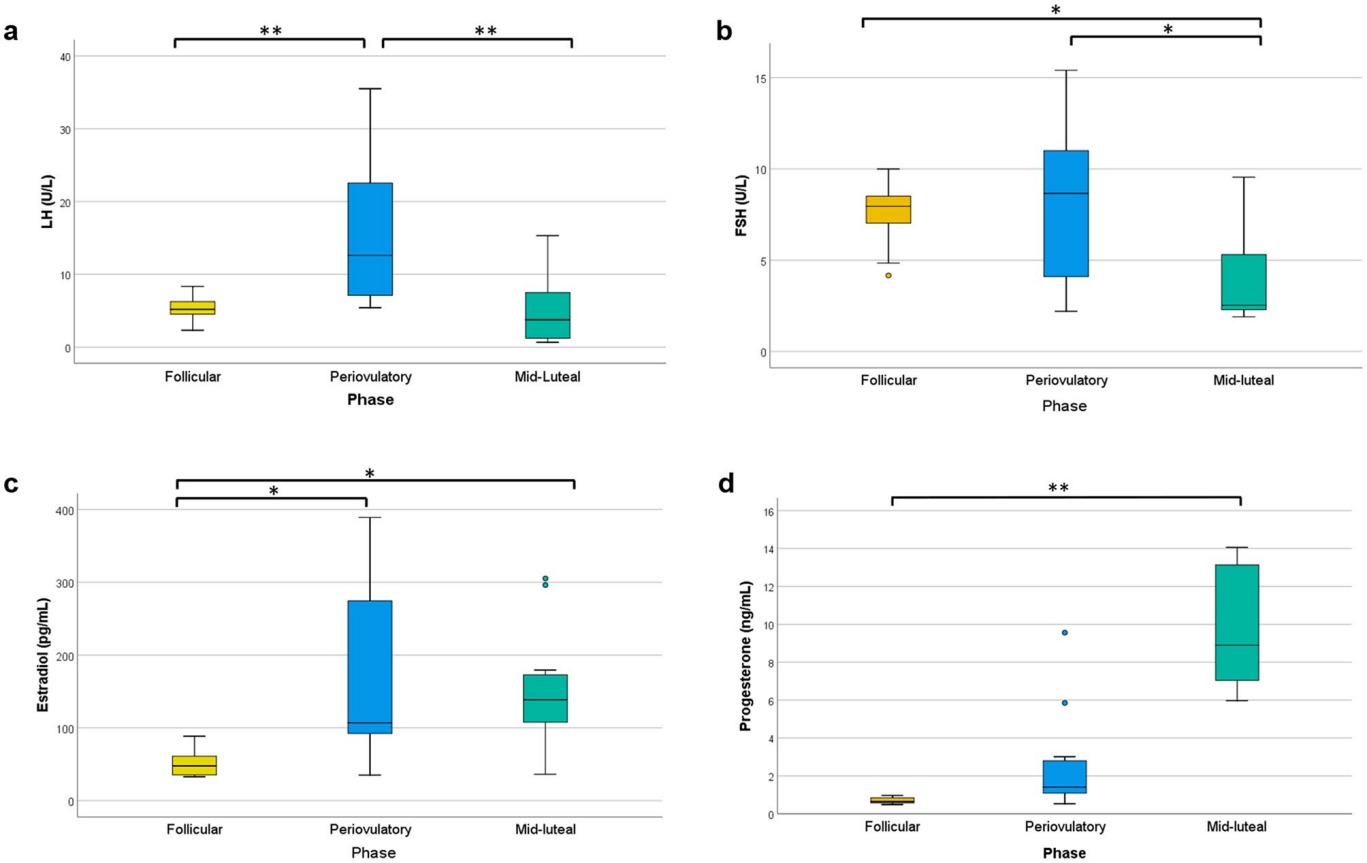
Serum hormone concentrations across the menstrual cycle. Box-and-whisker plots of (**a**) luteinizing hormone (LH), (**b**) follicle stimulating hormone (FSH), (**c**) estradiol and (**d**) progesterone levels, measured in follicular, periovulatory and mid-luteal phases (n = 11). Statistical differences were determined by Friedman test followed by Dunn-Bonferroni test.*p < 0.05, **p < 0.01.

The participants included in the study presented a normal pattern of sex hormone levels and profiles during the menstrual cycle (Table 1 and Fig. 1). Typical LH peak was found at periovulatory phase, compared to follicular (*p* = 0.009) and mid-luteal phases (*p* = 0.002) (Fig. 1a). FSH concentration at periovulatory phase was also significantly increased with respect to follicular and mid-luteal levels (*p* < 0.05) (Fig. 1b).With regard to estradiol, its characteristic profile was found, with a peak at periovulatory phase (*p* = 0.042, compared to follicular phase) and a secondary increase in the mid-luteal samples (*p* = 0.011, compared to follicular phase) (Fig. 1c). Finally, as Table 1 and Fig. 1d show, a rise in progesterone levels until 8.9 pg/mL was observed at mid luteal phase (*p* = 0.002, compared to follicular phase).

**Figure 2:**
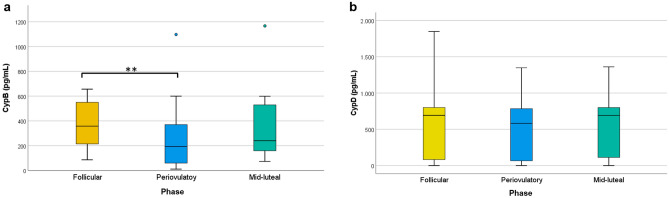
Concentration of cyclophilins B and D throughout the menstrual cycle. (**a**) Box-and-whisker plot of CypB concentrations in each phase. (**b**) Box-and-whisker plot of CypD concentrations in follicular, periovulatory and mid-luteal phases. Cyps concentrations were determined by ELISA kits (n = 11). Statistical differences were analysed with Friedman and Dunn-Bonferroni tests, **p < 0.01.

Regarding Cyps, a decrease of 49.9% (215 pg/mL) in CypB levels was found at periovulatory phase (Table 1 and Fig. 2a), compared to follicular phase (*p* = 0.009). In mid-luteal samples, CypB levels were recovered, showing a difference of 25.9 pg/mL (10.5%) with respect to the periovulatory concentrations. With regard to CypD, the pattern was similar, with a diminution of 16.0% (110.9 pg/mL) at periovulatory phase compared to follicular phase, and an increase of 109.6 pg/mL (15.9%) at mid-luteal phase, but differences between the phases were not significant (Table 1 and Fig. 2b).

Next, bivariate correlations among hormones and Cyps were analysed by Spearman coefficient in each phase. CypB levels showed a positive association with progesterone at follicular phase (Table 2). On the other hand, CypD concentrations presented a positive association with LH at periovulatory phase and with FSH levels at mid-luteal phase (Table 3).

**Table 2:** Bivariate correlations between hormone and cyclophilin B concentrations during the menstrual cycle. Correlations determined by Spearman coefficient (n = 11).*p < 0.05.

Variable/Phase	CypB		
	Follicular	Periovulatory	Mid-luteal
TSH	− 0.533 (*p* = 0.091)	− 0.136 (*p* = 0.689)	0.409 (*p* = 0.212)
FT4	− 0.155 (*p* = 0.649)	− 0.018 (*p* = 0.960)	− 0.027 (*p* = 0.937)
LH	0.036 (*p* = 0.915)	0.009 (*p* = 0.979)	0.009 (*p* = 0.979)
FSH	− 0.200 (*p* = 0.555)	− 0.273 (*p* = 0.446)	0.055 (*p* = 0.873)
Estradiol	0.231 (*p* = 0.521)	0.415 (*p* = 0.205)	− 0.391 (*p* = 0.235)
Prolactin	0.233 (*p* = 0.546)	0.200 (*p* = 0.606)	0.571 (*p* = 0.139)
Progesterone	0.657* (*p* = 0.039)	0.100 (*p* = 0.769)	− 0.370 (*p* = 0.293)

TSH, thyroid-stimulating hormone; FT4, free thyroxine; LH, luteinizing hormone; FSH, follicle-stimulating hormone; CypB, cyclophilin B.

Significant values are shown in bold.

**Table 3:** Bivariate correlations between hormone and cyclophilin D concentrations across the menstrual cycle. Correlations determined by Spearman coefficient (n = 11). *p < 0.05 and **p < 0.01.

Variable/Phase	CypD		
	Follicular	Periovulatory	Mid-luteal
TSH	− 0.468 (*p* = 0.147)	− 0.569 (*p* = 0.067)	0.064 (*p* = 0.852)
FT4	− 0.573 (*p* = 0.065)	− 0.308 (*p* = 0.387)	− 0.378 (*p* = 0.252)
LH	0.027 (*p* = 0.936)	0.743** (*p* = 0.009)	0.583 (*p* = 0.060)
FSH	− 0.274 (*p* = 0.474)	0.612 (*p* = 0.060)	0.633* (*p* = 0.036)
Estradiol	0.140 (*p* = 0.699)	− 0.050 (*p* = 0.883)	− 0.460 (*p* = 0.154)
Prolactin	0.351 (*p* = 0.354)	0.611 (*p* = 0.081)	0.252 (*p* = 0.548)
Progesterone	− 0.027 (*p* = 0.940)	− 0.400 (*p* = 0.223)	− 0.280 (*p* = 0.434)

TSH, thyroid-stimulating hormone; FT4, free thyroxine; LH, luteinizing hormone; FSH, follicle-stimulating hormone; CypD, cyclophilin D.

Significant values are shown in bold.

## Discussion

This study describes for first time the variations of CypB blood levels across a menstrual cycle. The protein presented maximum levels at follicular phase, followed by a decrease at periovulatory phase and a partial recovery at mid-luteal phase. The pattern found in CypB levels agrees with previous works in which pro-inflammatory mediators such as C-reactive protein and interleukin-6 presented increased concentrations during early follicular phase in both endometrium and serum, followed by a decrease in ovulation and an augmentation in luteal phase^20,21^.

These results suggest a potential role of CypB in the cyclic events that affect endometrium that could be related to the cyclic pattern observed in CD147 expression and with the regulation of matrix metalloproteases secretion^22^. Matrix metalloproteases levels increase after estradiol and progesterone drop just before menses, so the rise observed in CypB levels in mid-luteal phase could be augmenting CD147 expression and therefore, matrix metalloproteases release^23^. A correct remodelling is necessary for the preparation of endometrium for embryo implantation and pregnancy. In fact, CD147 has a role in implantation and its levels are reduced in women with reproductive problems^6,7^. Moreover, overexpression of the receptor has been reported in endometriosis, so the regulation of this transmembrane protein expression has a key role on the female reproductive system^24^. In this sense, the already known function of CypB in CD147 activation, as well as the fluctuation in blood levels across the menstrual cycle described in this work, point to a function of this protein in endometrium remodelling and fertility. In fact, it has been reported that CypA, another immunophilin that activates CD147, has a role in embryo implantation that is regulated by estradiol and progesterone^25^. Importantly, the fluctuations of CypB serum levels over the menstrual cycle should be taking into account in studies involving this protein. The inclusion of premenopausal woman must be handled carefully, as the menstrual cycle could affect to the outcomes of the study.

With respect to CypD, its blood levels presented the same trend than the observed in CypB, but statistical significance was not reached. Interestingly, a positive correlation of CypD with LH and FSH was found at periovulatory and mid-luteal phases, respectively. To our knowledge, this relationship among CypD, LH and FSH had not been previously described. On the other hand, despite the reported relationship among estradiol and TSH with CypD at cellular level, we did not find a correlation between their serum levels^10–12^. Regarding CypB, it was only correlated with progesterone at follicular phase. As this hormone presents basal concentrations in this phase, the correlation does not seem be of physiological importance. Intracellularly, CypB acts as a co-regulator of prolactin-induced genes, but we did not find an association among their blood levels^13^. Intracellular and extracellular Cyps have quite different functions. CypB has a key role in endoplasmic reticulum homeostasis but it also acts as a chemokine and induces the release of pro-inflammatory cytokines when it is released to the extracellular space^3^. This different role could be related to the lack of association of Cyps with hormones that had been reported to participate in their intracellular modulation.

Regarding biochemical parameters, we found significant fluctuations in glucose concentration throughout the menstrual cycle. The highest levels were detected at follicular phase, followed by a decrease at ovulation and a slight augmentation at the mid-luteal extraction, agreeing with previous works^26^. The same profile had been reported for TG and low-density lipoprotein in woman with regular cycle, whilst HDL levels were maximal in ovulation^27^. Our results of these metabolic parameters show the same profile, but we did not find significant differences.

This lack of statistical significance could be related to the small size of the population, which is the main limitation of the study. However, despite the small number of samples included, we do find significant differences in CypB concentrations across the menstrual cycle that reinforce the potential role of the protein in this physiological event.

## Conclusions

This study provides the first description of the variations in CypB levels throughout the menstrual cycle, opening a door for future works at clinical, cellular and molecular level to confirm the function of this Cyp in menstrual cycle. Moreover, the correlation of CypD levels with LH and FSH suggest an involvement of these hormones in the regulation of this immunophilin that should be explored.

## Data Availability

The datasets generated and/or analysed during the current study are available from the corresponding author on reasonable request.
